# Type distribution, viral load and integration status of high-risk human papillomaviruses in pre-stages of cervical cancer (CIN)

**DOI:** 10.1038/sj.bjc.6602648

**Published:** 2005-06-07

**Authors:** S Andersson, H Safari, M Mints, I Lewensohn- Fuchs, U Gyllensten, B Johansson

**Affiliations:** 1 Institute for Clinical Science, Division of Obstetrics and Gynaecology, Karolinska University Hospital, Huddinge, Karolinska Institute, 141 86 Stockholm, Sweden; 2 Division of Clinical Virology, Department of Laboratory Medicine, Karolinska University Hospital at Huddinge, Karolinska Institute, 141 86 Stockholm, Sweden; 3 Department of Genetics and Pathology, Section of Medical Genetics, Rudbeck Laboratory, University of Uppsala, 751 85 Uppsala, Sweden

**Keywords:** human papillomavirus, viral load, integration, type

## Abstract

A series of 176 archival cervical intraepithelial neoplasia (CIN) was analysed for the presence, viral load and integration status of ‘high-risk’ types of human papillomavirus (HR-HPV). The samples were assayed using newly developed methods based on real-time PCR. Two methods for the extraction of DNA from the paraffin-embedded biopsies were compared: a protocol based on the MagNA pure system (Roche) and a Qiagen spin column kit (Qiagen). It was possible to amplify 94% (166) of the samples. Of these, 36, 63 and 80% of the CIN I, II and III cases contained HR-HPV. HPV 16 was the most prevalent, and was found in 20, 28 and 46% of the CIN I, II and III cases, respectively. The second most frequent HR-HPV was type 33 group, and in CIN II it was as prevalent as HPV 16. The median number of copies of HR-HPV per cell was not significantly different in the CIN I, II and III cases, but there was a wide range of viral load values over several magnitudes, regardless of the grade of CIN. All samples were found to contain integrated forms of HPV 16, frequently mixed with an episomal form.

Intensive research has provided evidence for a central, causal role of certain types of HPV in the development of invasive cervical carcinoma (Schiffman *et al*, 1993; Davidson *et al*, 1994; Walboomers *et al*, 1999; Bosch and Munoz, 2002). The presence of high-risk HPV-DNA identifies both women with disease and those who are at a particular risk of progression to disease (Nobbenhuis *et al*, 1999). Persistent infection with high-risk types of HPV (HR-HPV), especially type 16, is regarded as the principal risk factor in the development of squamous cervical lesions or squamous cervical cancer (Munoz *et al*, 2003). However, most infections with HPV regress spontaneously, and, for the cases that do progress to cancer, a long period of latency is normally observed. HPV infections are prevalent and often transient among younger women, with a peak of 20–25% at 20–24 years of age. With increasing age, there is a decline in the prevalence of HPV to about 7% at 35 years of age (Soutter and Fletcher, 1994). It is likely that HPV-positive women at that age represent a subset of individuals who do not manage to clear their infections spontaneously. Thus, the persistence of a high-risk HPV infection is associated with the risk of developing cervical intraepithelial neoplasia (CIN).

A high prevalence of transient infections makes detection of the virus an inefficient means of identifying women at risk of developing cervical cancer (Ho *et al*, 1998; Jacobs *et al*, 2000). Viral load has been suggested as a marker of nontransient infection; a high HPV load in archival Pap smears with normal cytology had a considerably higher risk of developing carcinoma *in situ*, and even cervical squamous carcinoma (Josefsson *et al*, 2000; Lorincz *et al*, 2002; Dalstein *et al*, 2003; Gravitt *et al*, 2003; Moberg *et al*, 2004). Recently, it has been concluded that HPV load is a type-dependent risk marker for invasive carcinoma (Moberg *et al*, 2005). Furthermore, it has been suggested that there is an association between a high viral load and the persistence of infection with HPV (Ho *et al*, 1998).

The integration status of high-risk HPV in premalignant cervical lesions might be a further promising risk marker for progression of cervical cancer (Lazo, 1997; Kalantari *et al*, 1998). Viral DNA integration into the host cell genome usually disrupts the E1 and E2 open reading frames, while those of E6 and E7 remain intact (Kalantari *et al*, 1998, 2001). The deletion of E2 ORF, due to the integration, results in disruption of expression of E2 protein and subsequent upregulation of the transcription of the oncogenic E6 and E7 proteins (Romanczuk and Howley, 1992). Continuous production of oncogenic E6 and E7 proteins contribute to the malignant state in infected tissue. Thus, viral integration into the host genome is possibly another critical event for malignant transformation.

In the present study, our aim was to evaluate the frequency of HR-HPV types and viral load in a series of paraffin-embedded biopsies with pre-neoplastic and neoplastic lesions. Based on these data, we sought to determine if type distribution and viral loads of different HR-HPV types in CIN I–III lesions show any significant differences. We also evaluated any differences in the integration status of HPV 16, as a function of the grade of CIN.

## MATERIALS AND METHODS

Specimens from 176 biopsies, which represented CIN I–III lesions, obtained between 2002 and 2003, were collected from the Department of Pathology, Karolinska University Hospital at Huddinge. The biopsies were fixed in neutral buffered formalin and embedded in paraffin. Serial sections were cut from each block. The first was stained with haematoxylin-eosin and evaluated histopathologically, whereas the following were used for the preparation of DNA and the HPV test. The biopsies were grouped according to the morphological diagnosis and types of HPV present. The diagnosis of all cases was reviewed and reconfirmed by one pathologist.

### Extraction of DNA from tissue sections

To optimise the DNA extraction method for our study, two different DNA-extraction protocols were compared.

#### Extraction using specific magnetic glass particles (MGPs) to bind DNA (Roche MagNA pure kit)

Protocol A is based on binding the isolated DNA from tissue samples to the surface of specific MGPs. This procedure is automated and performed by using the *MagNA Pure LC* system (*Roche*).

The paraffin-embedded (formaldehyde-fixed) tissue sections were deparaffinised with xylene and absolute ethanol. To melt the paraffin, the paraffin-embedded tissue sections were incubated at 65°C for 10 min, and, subsequently, 1 ml xylene and 500 *μ*l absolute ethanol were added and mixed gently. After centrifugation at 14 000 rpm for 2 min, the supernatant was discarded and 1 ml absolute ethanol was added. After a new 2-min centrifugation at 14 000 rpm, the pellet was dried for 10–15 min using a Speed Vac vacuum drier.

The dried pellet was re-suspended in a 130 *μ*l tissue lysis buffer and 20 *μ*l proteinase K, and was subsequently incubated at 56°C overnight. The cell suspension was mixed with a 350 *μ*l cell lysis buffer in a sample cartridge, and then loaded into the *MagNa Pure* instrument. The other reagents, that is, MGPs, which bind the total DNA from the lysed cell suspension, proteinase K, and a wash and elution buffer, were also loaded into the instrument. The extracted DNA was eluted with a 50 *μ*l elution buffer.

#### Extraction using a specific spin column to bind DNA (Qiagen kit)

Protocol B is a manual DNA extraction method, which is based on the QIAamp Spin Column kit (Qiagen).

The paraffin-embedded tissue sections were deparaffinised by the procedure described above. After addition of 1 ml xylene, the tube was incubated at room temperature for 30 min and subsequently centrifuged at 14 000 rpm for 5 min. This step was repeated once, after which 1 ml absolute ethanol was added to the tube, followed by centrifugation at 14 000 rpm for 5 min. The pellet was dried in the Speed Vac for 10–15 min and was then resuspended in a 180 *μ*l tissue lysis buffer and 20 *μ*l proteinase K. After incubation of the solution at 56°C overnight, a 200 *μ*l cell lysis buffer was added. A new incubation at 70°C for 10 min was carried out before the addition of 200 *μ*l 95% ethanol. The mixture was then applied to the QIAamp spin column. After centrifugation of the column at 8000 rpm for 1 min, the column was washed twice with a 500 *μ*l wash buffer AW1 followed by AW2. The purified DNA was eluted with 50 *μ*l distilled water.

### Detection and quantification of HPV by the ‘QUANTOVIR HPV’ system

Extracted DNA from the MagNA pure was subsequently quantified, and HR-HPV typed, by using the ‘QUANTOVIR HPV’ detection and quantification system as described by Moberg *et al* (2003).

The ‘QUANTOVIR HPV’ detection and quantification kit was used for preparation of the Master Mix. The real-time PCR reaction was performed in a total volume of 25 *μ*l (22 *μ*l Master Mix and 3 *μ*l DNA template), and with the use of optical 96-well micro-plates were used. The Master Mix contained 1 × PCR buffer – 200 *μ*M each of dATP, dCTP and dGTP and 400 *μ*M dUTP – 3.5 mM MgCl_2_, 10 × G-carboxy-X-rhodamine,6-Rox, and 0.625 U Taq DNA polymerase final concentrations in sterile water. The PCR amplification program consisted of an initial holding step of 10 min at 95°C, followed by a two-step cycle of 15 s at 95°C and 1 min at 57°C for 40 cycles. Three parallel reactions per sample were performed: no. 1 quantified HPV 16, 31, 18 and 45 (the last two together); no. 2 quantified HPV 33, 35, 39, 52, 58 and 67 (HPV 33, 52, 58 and 67 together); no. 3 quantified the single copy human gene for hydroxymethylbilane synthase. A total of six nontemplate control reactions, which consisted only of PCR components without template DNA, were used as negative controls to ensure that the reagent mix was free of contamination. The assay was performed by using the ABI PRISM 7700 Sequence Detection System, running the 1.6.3 version of the SDS program (Applied Biosystems).

Since the ABI 7700 software could not quantify three different fluorophores in the same reaction, a separate QUANTOVIR algorithm was developed, using the MATLAB program vers. 5.2.1, directly coupled to the data collection program of the ABI 7700 apparatus. This was used for calculation of the threshold cycle, and conversion of the data into HPV copy numbers per cell. The number of cells was calculated from the copy numbers of a single copy human gene (hydroxymethylbilane synthase), and this and other calculations were performed automatically by the specially developed software.

The established linear dynamic range of the method is 10^2^–10^7^ copies of HR-HPV per genomic DNA equivalent (Moberg *et al*, 2003).

### HPV integration status

The recently developed real-time PCR-based integration method of Peitsaro *et al* (2002) was performed by using the ABI prism 7700 Sequence Detection system and the TaqMan PCR Master Mix (PE Applied Biosystems, Perkin-Elmer). The basic principle of the method is to use specific primers and probes for separate assays of the region (‘hinge region’) of the E2 ORF, most often deleted during integration, and part of the E6 ORF. The latter ORF is always retained in the integrated virus. The E6 probe was labelled with FAM at the 5′- and TAMRA at the 3′-end, while the E2 probe was labelled with VIC at the 5′- and TAMRA at 3′-end. The final concentrations of primers and probes in a total of 50 *μ*l of reaction mixture were 0.3 and 0.1 *μ*M, respectively. Two standard curves for E6 and E2 were obtained by amplification of a dilution series of one million to 10 copies of a clone of the entire HPV 16 genome in pBR322. There was a linear relationship between the threshold cycle values plotted against the log of the copy number over the entire range of dilutions (data not shown). The 96-well optical plate was used to run reactions. In each experiment, samples were loaded in duplicate and three negative controls were included.

DNA from the SiHa cell line, containing one copy of fully integrated HPV 16, was also used as a control for full integration in these experiments. No E2 signal should be produced, since the assayed region of E2 is deleted in SiHa cells.

## RESULTS

The study comprises 166 cases: among these 50 (30%) CIN I, 60 (36%) CIN II and 56 (34%) CIN III. The mean age of women with CIN I was 39.6, in women with CIN II was 33.3 and in women with CIN III was 34.7.

### Extraction method

A comparison between the Qiagene and Magna Pure kits showed that Magna Pure provided higher DNA yields than Qiagene for the extraction of paraffin-embedded tissue sections. The *MagNA* pure system has further advantages in its semi-automation and quickness. It has been used throughout this study.

### Frequencies of HR-HPV

**Figure 1:**
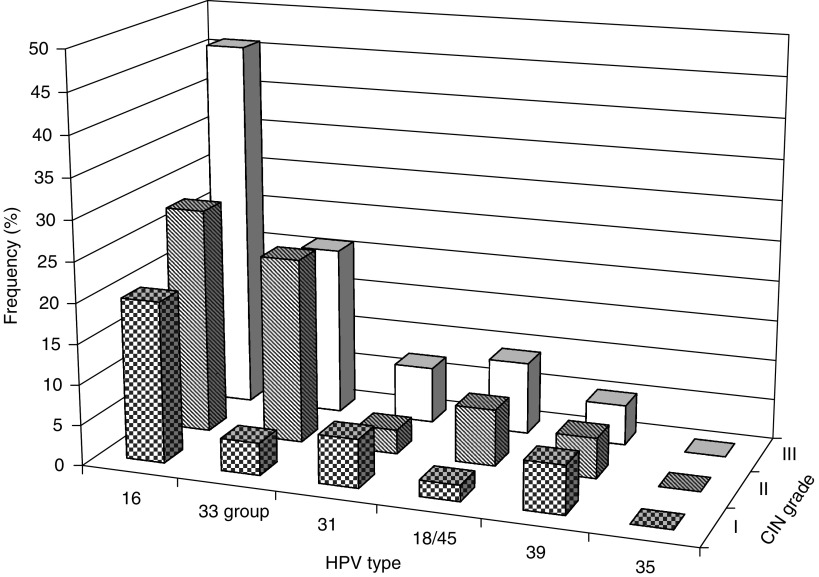
Frequencies of the HR-HPV types in samples from different CIN grades. Frequencies of the different types of HR-HPV in total 166 samples, distributed over the three CIN grades, are shown. CIN I – chequered bars, CIN II – striped bars, CIN III – blank bars.

Of the 176 samples analysed with the ‘Quantovir HPV’ system, 10 were totally negative, that is, no signal was obtained from the human gene. The frequencies of the different types of HR-HPV in the remaining 166 samples, from different stages of CIN, are summarised in Table 1 and Figure 1.

There was a steady increase in the frequency of HR-HPV from CIN I to III. Of the CIN III samples, 45 (80%) were positive for HR-HPV. As shown in Figure 1, the increased frequencies of HR-HPV were due to increase in the number of positive cases with HPV 16, 18/45 and 33 groups (HPV 33, 52, 57, 58), while the number of positive cases with HPV 31 and 39 was constant. As could be expected, the oncogenic HPV 16 predominated at all stages of CIN: 20, 28 and 46% at CIN I, II and III, respectively.

However, the high proportion (23%) of the HPV 33 group in the biopsies from CIN II seemed almost as high as that for HPV 16 (Table 1, Figure 1). No HPV 35 was found in this material.

### Viral loads

#### HR-HPV

**Figure 2:**
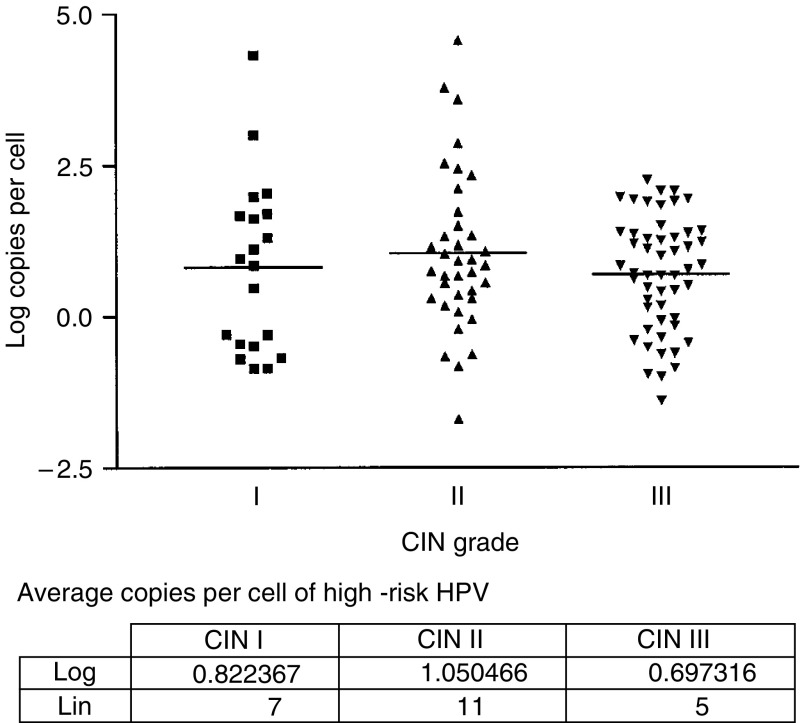
HR-HPV viral load and CIN grade. Distribution of the log values for viral load at different CIN grades is shown. The horizontal line denotes average log values. The table contains the log as well as line values for average number of HR-HPV copies per cell at the different CIN grades.

The viral loads obtained for HR-HPV in the different grades of CIN are summarised in Figure 2. There was a distribution of viral load values of several orders of magnitude at all grades of CIN. The span of values of copies of HR-HPV per cell ranged from 0.14 to 8400. The mean values of the copies per cell were: CIN I – 8; CIN II – 17 and CIN III – 6. However, by using the one-way ANOVA analysis, there was no significant difference determined between these mean values.

### HPV 16

**Figure 3:**
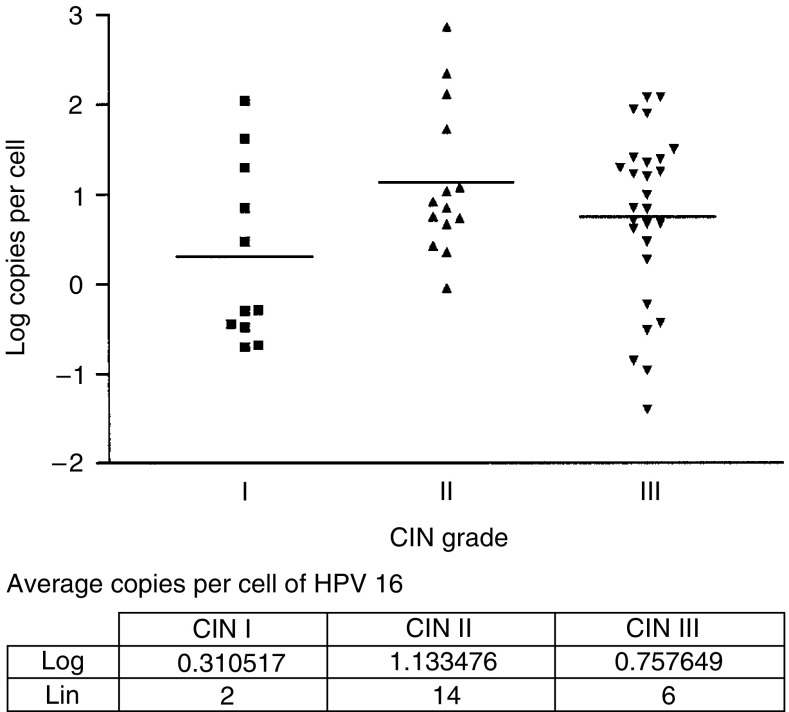
HPV 16 viral load and CIN grade. Distribution of the log values is shown for viral load at different CIN grades. The horizontal line denotes average log values. The table contains the log per cell at the different CIN grades.

Since HPV 16 is the most predominant type in this material, as well as in cervical squamous carcinomas, viral load values were examined separately for this type. As for HR-HPV in general, there was an equally broad distribution of viral loads (Figure 3). The mean values of copies per cell were: CIN I – 2, CIN II – 18 and CIN III – 6. Moreover, in this case, there was no significant difference between the mean values as determined by the one-way ANOVA analysis.

### Viral integration

**Figure 4:**
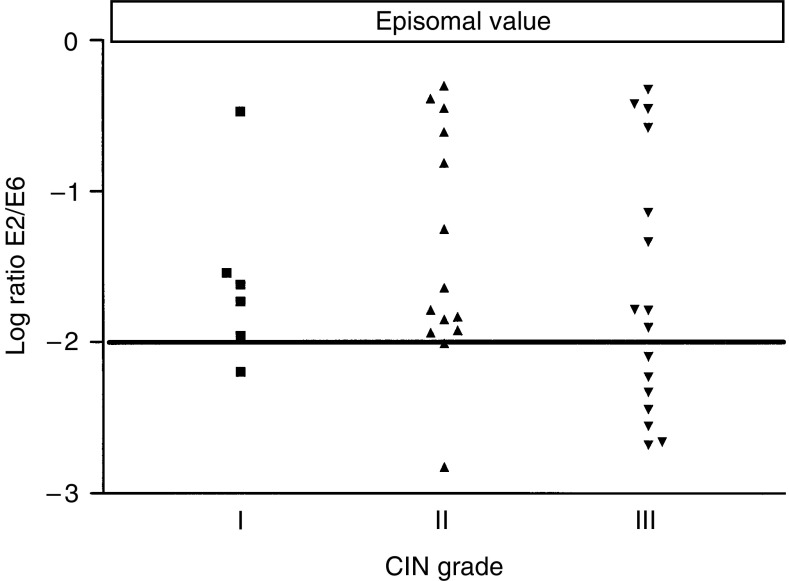
HPV 16 integration status at different CIN grade. Integration status is calculated from the ratios of copy numbers for gene *E2* divided by the same values for gene *E6*. The E2 values are related to the number of episomal copies, whereas the E6 values arise from the total number of HPV 16 copies. The values are shown in log form. The top horizontal bar indicates the log ratio expected from purely episomal form (E2/E6 ratio=1). The horizontal line in the upper graph at log −2, indicates an integration status, where there is one episomal form for a total of 100 copies of HPV 16.

A specially designed real-time PCR method (Peitsaro *et al*, 2002) was used to estimate HPV 16 integration by comparing viral loads from the parts of the E2 ORF that are mostly deleted during integration, and those of the E6 ORF. The latter gives an estimate of the total number of HPV 16 genomes, episomal and integrated. All of the 48 HPV 16 cases, found in the previous typing and viral load determination, were found to contain integrated virus genomes. However, there was a higher proportion of cases with no detectable copies of the *E2* gene, indicating complete integration (Table 2), at the CIN III stage (36%) than at the CIN I (25%) and CIN II (7%) stages. Among the cases showing E2/E6 ratios less than 1.0, presumably containing episomal and integrated virus genomes, the relative contents of the two forms varied considerably (Figure 4). The average ratios of episomal and integrated forms were not significantly different in the three stages of CIN, as determined by the one-way ANOVA analysis. However, if a cutoff value is introduced at a ratio of 1 : 100 of episomal and integrated forms, respectively, there are significantly more cases below this value in CIN III (horizontal line in Figure 4).

## DISCUSSION

Epidemiological and molecular biological studies have shown that infection with high-risk HPV is the most important aetiological agent in the pathogenesis of cervical cancer (Bosch *et al*, 1995). At present, more than 99% of the squamous cell tumours are considered to harbour oncogenic types of HPV (Walboomers *et al*, 1999). HPV 16, 18, 45, 31 and 33 are the most frequently identified viruses in CIN II–III and cervical squamous cell carcinomas (Munoz *et al*, 2003).

In our study, a stored formalin-fixed and paraffin-embedded material was used for the Quantovir test. We investigated the presence of different types of HPV and the viral loads in women with CIN I–III. The Quantovir test, a quantitative assay which is applicable for clinical use and capable of detecting a range of high-risk types of HPV involved in cervical carcinogenesis, has been previously described in detail (Moberg *et al*, 2003). The system has many technical advantages: a high sensitivity, high specificity and wide dynamic range. Quantification of three different fluorophore molecules in each reaction tube is possible by using a newly developed software to calculate copies of HPV-DNA per cell. Identification and a viral load estimate of HPV types in a reaction involving other types is reliable as long as it represents at least 1–10% of the amount of the main HPV type (Moberg *et al*, 2003). The sensitivity of the assay decreased when the ratio between the types of HPV was less than 1 : 100. Furthermore, several techniques for analysis of HPV are available, causing variations with respect to both sensitivity and specificity. Although the linear dynamic range of the method was certified only down to 100 copies HR-HPV per cell equivalent (Moberg *et al*, 2003), compared to the five copies described by Gravitt *et al* (2003), quantification was also possible below that level. However, these figures should be regarded as approximative. We compared two extraction methods the MagNA pure system (Roche) and a Qiagen spin column kit (Qiagen). We found that the MagNA pure kit was more sensitive and provided the highest DNA yields from the material.

By using the Quantovir test, we identified five different oncogenic types of HPV (16, 31, 18/45, 33, 39) in our material. As could be expected, the oncogenic HPV 16 predominated at all stages of CIN: 20, 28 and 46% at CIN I, II and III, respectively. The most obvious findings were that HPV types 31 and 39 did not occur more frequently in higher degrees of CIN, and that the frequency (23%) of the HPV 33 group in the biopsies from patients with CIN II seemed almost as high as that for HPV 16. In previous studies, in which the sensitive PCR technique for detection and typing of HPV DNA was used, it was reported that HPV 16 is the most common type, followed by types 31 and 18. Even in a general population of 32–38-year-old healthy women, HPV 16 is the most prevalent type (2.2%) (Forslund *et al*, 2002).

Earlier studies have described the relative frequency of the most common oncogenic types of HPV in pre-invasive cervical lesions and cervical carcinoma. Our results demonstrated a predominance of HPV 16, followed by HPV 33. However, it is also of importance to identify the less prevalent and rare types of HPV in different pre-invasive lesions since the types of HPV identified may vary geographically.

Several studies have published reports investigating viral load's association with risk for cervical cancer and its precursors. The majority use HCII to measure viral load, and while some find viral load to be positively associated with increased risk for prevalent or incident disease (Sun *et al*, 2001, 2002; Dalstein *et al*, 2003) others do not (Lorincz *et al*, 2002; Sherman *et al*, 2002). Recent studies employing quantitative PCR to estimate HPV load show association between viral load and prevalent or incident disease more consistently (Ylitalo *et al*, 2000; Gravitt *et al*, 2003; Schlecht *et al*, 2003). Considerable viral-load variation has been observed within histopathological grades of the disease (Swan *et al*, 1999), making it hard to define uniform cutoff values. In addition, viral loads have been reported to be associated with lesion size rather than lesion severity.

It is noteworthy that in our study this is the first time that viral load of HR-HPV has been estimated in a systematic manner in CIN I–III lesions. As we expected and as previous studies have shown, there was a wide range of viral load values over several magnitudes, regardless of CIN grade (Peitsaro *et al*, 2002), but the average of copies of HR-HPV per cell were not significantly different in the CIN I, II and III cases (8, 17 and 6, respectively). The same was found also for the separately calculated values of HPV 16. The results might be influenced by the sample material, which was paraffin-embedded, formaldehyde-fixed biopsies. In general, it is difficult to obtain high DNA yields from such material as compared with fresh biopsies.

In accordance with a previous publication (Peitsaro *et al*, 2002), integration was detected at all stages of CIN. A real-time PCR-based integration assay was employed, where specific primers and probes directed to a unique region of E2 ORF and E6 ORF, respectively, were used. The hinge region of E2 ORF, which is most often deleted during HPV 16 integration into the host genome (Kalantari *et al*, 1998), was selected to use as target to design E2 primers and probes.

Our results showed that all HPV 16 cases (totally 48) were found to contain integrated virus genome. However, there was a higher proportion of cases with complete integration at the CIN III grade (nine cases) than at CIN I and II (2 and 1 cases); the average ratio of (copy numbers of E2 gen/copy numbers of E6 gen) was not significantly different in the three CIN grades. The reason could be, for instance, the limited number of samples (48 samples), or the episomal signal could arise from border areas of the biopsy that are not yet malignant. If a microdissection could be performed, to select only the parts of the biopsy with clear malignant properties, it is possible that the difference in the integrated/episomal ratio would be more significant.

As we expected, there was a significantly higher number of CIN III samples showing very low levels of episomal compared to integrated form (<1/100), than for the other CIN grades.

A combination of organised and opportunistic screening has reduced the incidence of squamous carcinoma substantially during the last decades in Sweden (Mählck *et al*, 1994; Ponten *et al*, 1995). In several studies, the Pap smear has been shown to have a high false-negative rate (Macgregor *et al*, 1994). A review of evidence-based data revealed that as many as 50% of precancerous cervical lesions may be missed by a single Pap test (Sherman *et al*, 1994). Another drawback of cervical cytology is its high false-positive rate (Sherman *et al*, 1994). Kinney *et al* (1998) showed that only 30% of women with histologically confirmed high-grade disease had corresponding atypia in their Pap smears. Studies have shown that 15–28% of HPV-DNA-positive women with normal cytology develop CIN within 2 years, compared with only 1–3% of HPV-DNA negative women (Schiffman and Castle, 2003). Against this background, a new screening strategy for cervical cancer has been presented in which HPV testing is combined with cytological examinations (Walboomers *et al*, 1999). In addition, the abundance of transient infections and lack of treatment for HPV infections make it impractical to follow up all infected individuals. HPV testing was identified to have higher sensitivity and equal specificity compared to repeated Pap smear collection as a triage for CIN II–III (Andersson *et al*, 2004). Viral load and integration have been proposed, as ways of increasing the specificity of HPV tests. Based on others results (Ylitalo *et al*, 2000; Moberg *et al*, 2004), women infected with a high viral load of HPV 16 are at increased risk of developing cervical carcinoma *in situ* already several years before diagnosis, compared to women infected with a low viral dose. Viral load mainly seems to have relevance for HPV 16-related tumours. By using quantitative methods to measure HPV viral load, we probably have a sensitive tool to identify women of over-risk for cervical carcinoma. This would have important applications for the cervical screening. Prospective randomised studies including quantitative HPV testing are needed on fresh material.

Vaccines emerging today are directed mainly against HPV 16 and 18. Even if vaccines were expanded to cover HPV 31 and 45, they would still only prevent 80% of cervical cancers worldwide. HPV vaccination is likely to significantly reduce HPV infection and cervical cancer burden, but the need for screening of some form is likely to remain in the foreseeable future.

## Figures and Tables

**Table 1:** Frequency of HR-HPV types in samples from different CIN grades

	**CIN grade**		
**Frequency, % of DNA-positive samples**	**I**	**II**	**III**
HR-HPV	36	63	80
HPV 16	20	*28*	46
HPV 33 group	4	*23*	21
HPV 18/45	2	7	9
HPV 31	6	*3*	7
HPV 39	6	5	5
HPV 35	0	0	0

CIN=cervical intraepithelial neoplasia. Total number of human DNA-positive samples (166) were: CIN I, 50; CIN II, 60; CIN III, 56.

**Table 2:** Number of totally integrated HPV 16 in samples from different CIN grades

	**CIN grade**			
	**I**	**II**	**III**	**Totally**
Number of samples	8	15	25	48
Totally integrated	2	1	9	12
Totally int. (%)	25	7	36	
